# Chromatinization of the KSHV Genome During the KSHV Life Cycle

**DOI:** 10.3390/cancers7010112

**Published:** 2015-01-14

**Authors:** Timsy Uppal, Hem C. Jha, Subhash C. Verma, Erle S. Robertson

**Affiliations:** 1 Department of Microbiology and Immunology, School of Medicine, University of Nevada, 1664 N Virginia Street, MS 320, Reno, NV 89557, USA; E-Mails: tuppal@medicine.nevada.edu (T.U.); scverma@medicine.nevada.edu (S.C.V.); 2 Department of Microbiology and the Tumor Virology Program of the Abramson Cancer Center, Perelman School of Medicine at the University of Pennsylvania, 201E Johnson Pavilion, 3610 Hamilton Walk, Philadelphia, PA 19104, USA; E-Mail: hemjha@mail.med.upenn.edu

**Keywords:** KSHV, oncogenic virus, LANA, RTA, epigenetics, viral chromatin, histone modifications, DNA methylation, PAN RNA, *de novo* infection

## Abstract

Kaposi’s sarcoma-associated herpesvirus (KSHV) belongs to the gamma herpesvirus family and is the causative agent of various lymphoproliferative diseases in humans. KSHV, like other herpesviruses, establishes life-long latent infection with the expression of a limited number of viral genes. Expression of these genes is tightly regulated by both the viral and cellular factors. Recent advancements in identifying the expression profiles of viral transcripts, using tilling arrays and next generation sequencing have identified additional coding and non-coding transcripts in the KSHV genome. Determining the functions of these transcripts will provide a better understanding of the mechanisms utilized by KSHV in altering cellular pathways involved in promoting cell growth and tumorigenesis. Replication of the viral genome is critical in maintaining the existing copies of the viral episomes during both latent and lytic phases of the viral life cycle. The replication of the viral episome is facilitated by viral components responsible for recruiting chromatin modifying enzymes and replication factors for altering the chromatin complexity and replication initiation functions, respectively. Importantly, chromatin modification of the viral genome plays a crucial role in determining whether the viral genome will persist as latent episome or undergo lytic reactivation. Additionally, chromatinization of the incoming virion DNA, which lacks chromatin structure, in the target cells during primary infection, helps in establishing latent infection. Here, we discuss the recent advancements on our understating of KSHV genome chromatinization and the consequences of chromatin modifications on viral life cycle.

## 1. Introduction

Kaposi’s Sarcoma (KS), first described in 1872 by the Hungarian dermatologist Moritz Kaposi, is defined as a multiple idiopathic sarcoma of the skin. The causative agent of Kaposi’s sarcoma was identified as the human herpesvirus 8 (HHV8), or Kaposi’s sarcoma-associated herpesvirus (KSHV) from the tissue biopsies of AIDS-associated KS by Chang and Moore in 1994, using the representational difference analysis approach [1]. KSHV is an oncogenic γ-herpesvirus that establishes life-long persistent infection and causes tumors in immunosuppressed individuals; particularly, in transplant recipients and patients infected with HIV. Since its initial discovery in KS lesions, KSHV has been tightly linked with endothelial tumors, Kaposi’s sarcoma and two B-cell lymphoproliferative disorders, primary effusion lymphoma (PEL), also known as body cavity-based lymphoma [2], and a plasmablastic variant of multicentric Castleman’s disease (MCD) [3]. Additionally, KSHV has been linked to different lymphomas, including Burkitt’s lymphoma, Germinotropic Lymphoproliferative Disorder (GLD), multiple myeloma, angio-sarcomas, malignant skin tumors, angio-immunoblastic lymphoma and primary pulmonary hypertension [4,5,6]. There have also been reports of a new KSHV/HHV8-associated germinotropic lymphoproliferative disorder in HIV-seronegative individuals [7].

### 1.1. Clinical Diseases Associated with KSHV Infection

#### 1.1.1. Kaposi’s Sarcoma (KS)

Kaposiʼs sarcoma is the first tumor to be associated with HIV infection and remains the most common cancer in Sub‐Saharan Africa and the second most common cancer in HIV‐infected patients [8]. KS is a highly vascular, non-classical tumor of endothelial lymphatic origin clinically characterized by dark red, brown or purple patches or plaques found cutaneously, mucosally or viscerally [9]. KS lesions are characterized by the presence of spindle shaped, poorly differentiated and highly proliferative cells and by infiltration of inflammatory cells and extensive neo-angiogenesis [10]. Several studies indicate that KS spindle cells are of endothelial lineage as they express the vascular endothelial cell markers CD31, CD34, CD36 [11]. More recently, KS spindle cells are thought to be of lymphatic endothelial cell (LECs) origin because they express LYVE-1, VEGFR-3 and podoplanin, markers of the lymphatic endothelium, making it difficult to identify the precursor cell type [12,13]. KSHV is required for the development of KS with greater than 95% of KS lesions harboring KSHV viral DNA in latent phase [14]. The role of KSHV in KS development is complex and involves both latent and lytic genes, many of which are pirated versions of cellular genes. The virus is found in all epidemiologic-clinical forms of the disease, including classic, African (endemic), HIV-associated (epidemic) and iatrogenic (transplant associated) KS (reviewed in [15]). Classic KS (indolent form) usually is present as lesions in the lower and upper extremities without the involvement of lymph nodes and internal organs [16]. Endemic KS, on the other hand, can be indolent or aggressive and transplant-related that represents a relatively indolent and chronic condition with a rapidly progressive course involving lymph nodes, mucosa and inner organs [17]. HIV-related KS is the most frequent and aggressive form that includes the lymph nodes and visceral spreading, though the role of HIV infection in KS development is not very well understood [18]. Since the introduction of highly active antiretroviral therapy (HAART), there has been a decline in KS incidence. However, Kaposiʼs sarcoma continues to be diagnosed in HIV-infected patients.

#### 1.1.2. Primary Effusion Lymphoma (PEL)

PEL, also referred to as body cavity-based lymphoma (BCBL), is an aggressive form of non-Hodgkin’s B-cell lymphoma and is linked to KSHV infection and commonly found in late stage immunocompromised AIDS patients [19]. Evidence for the contributing role of KSHV in PEL development is attributed to detection of approximately 50–150 copies of KSHV latent genomes per cell with less than 1% of cells entering the lytic cycle [20,21]. Additionally, PEL cell survival has been shown to depend on the expression of CD138/syndecan-1, the KSHV latent genes (primarily ORF73, LANA) and viral microRNAs, highlighting the association between KSHV infection and PEL development [22]. Approximately 50% of PEL patients are KSHV-positive and found to be co-infected with Epstein-Barr virus (EBV), although cases of KSHV and EBV-negative PEL have also been reported [23,24]. Consistent growth of PEL cell lines in culture and easy induction of infectious KSHV virions release have made them a valuable *in vitro* infection model for understanding the molecular mechanisms of KSHV-related oncogenesis, in terms of cell transformation, signaling, cell growth, cell survival, angiogenesis and host-immune invasion, though how KSHV directly contributes to this B-cell malignancy is yet to be known [25].

#### 1.1.3. Multicentric Castleman’s Disease

The plasmablastic variant of Multicentric Castleman’s disease (MCD) containing large plasmablastic cells is frequently linked to KSHV infection and is usually characterized by lymphadenopathy and immune deregulation [14,26]. MCD is a lymphoproliferative disorder that is often diagnosed in HIV-infected patients. KSHV co-infection is predominantly observed in the immunoblastic B cells within the mantle zone of germinal centers of lymph nodes in almost all HIV-seropositive MCD cases and in less than 40% HIV-seronegative MCD cases [27]. Interestingly, in contrast to KS and PEL, KSHV infection in MCD is mostly lytic and thought to be driven by an elevated levels of cytokines, interleukin-6 and 10 (IL-6, IL-10) and the vascular endothelial growth factor (VEGF) [28]. Additionally, so far the co-infection of KSHV with EBV has not been detected in MCD plasmablasts [29].

### 1.2. Epidemiology and KSHV Transmission

Epidemiology studies on KSHV have identified eight distinctive subtypes based on the sequence analysis of a well-conserved minor capsid gene, ORF26 [30]. Though large parts of the KSHV genome are conserved in these variants, several regions were shown to be highly variable, including the K1 and K15 gene regions. The sequence variability of the K1 gene has led to the identification of seven major viral subtypes, namely A, B, C, D, E, F and G [31,32]. General nucleotide differences between these subtypes are only 3%, however up to 60% sequence variation is observed in the two-hypervariable regions (VR1 and VR2) of the K1 gene [33]. Only two of the KSHV subtypes, A and C have diversity in the region surrounding K15 with variants designated as P (or prototype), M (or minority), N and Q. These variations are thought to arise from a recombination event with an unknown progenitor herpesvirus [34,35].

KSHV prevalence is found to correlate with geographic location. For instance, subgroup A1–4 and subtype C are predominant among individuals in North Europe, the United States and in some regions of Asia and Middle East. Subgroup B1–4 is located primarily in sub-Saharan Africa while D and E strains are found principally in Australia, the Pacific and Brazilian Amerindians [36,37,38]. Several serology studies have indicated that KSHV infection is widespread in sub-Saharan Africa and the Amazon basin where greater than 50% population is infected [39]. Intermediate levels of KSHV prevalence are seen in Mediterranean, Middle East and the Caribbean regions where 4%–45% population is tested as KSHV-seropositive. Lower levels of viral infection occur in Northern Europe and North America with KSHV seropositivity ranging from 3% to 10% [40,41].

Although, the transmission modes and the risk factors for KSHV infection are not well understood, the virus is reported to transmit through both sexual and non-sexual transmission routes [42,43]. In low prevalence areas, a direct link has been reported between the number of sexual partners and the risk of KSHV infection, indicating sexual transmission as the predominant transmission route [44]. Also, seroprevalence is lower for women and children than in men. The epidemiology differs significantly in the high prevalence areas with equal seroprevalence amongst children, adult men and women highlighting non-sexual routes of virus transmission. Studies in Italy, northern Sweden and Uganda suggest that the KSHV virions are mostly transmitted via saliva and sometimes through water and insect bite indicating KSHV transmission is dependent on a combination of both environmental and genetic factors [45,46,47].

### 1.3. KSHV Genome and Its Chromatinization

KSHV has a double stranded DNA (dsDNA) genome of 165–170 kb consisting of long unique coding region, which is ~140 kb in length and is flanked with highly GC-rich 801 bp long terminal repeat sequences (TRs) that encodes for nearly 86 viral open-reading frames (vORFs), 12 miRNAs and a number of non-coding RNAs (ncRNAs) and antisense RNAs [48,49]. The viral genes encoded by KSHV can be classified into three categories: (1) Herpesviruses-common genes; (2) KSHV-unique genes, and (3) Cellular-homologous genes, which may include categories (1) and (2) [50]. Studies on KSHV virion-associated proteins indicate that the virus particle is formed through several highly specific protein-protein interactions among capsid, tegument proteins and glycoproteins [51].

**Figure 1:**
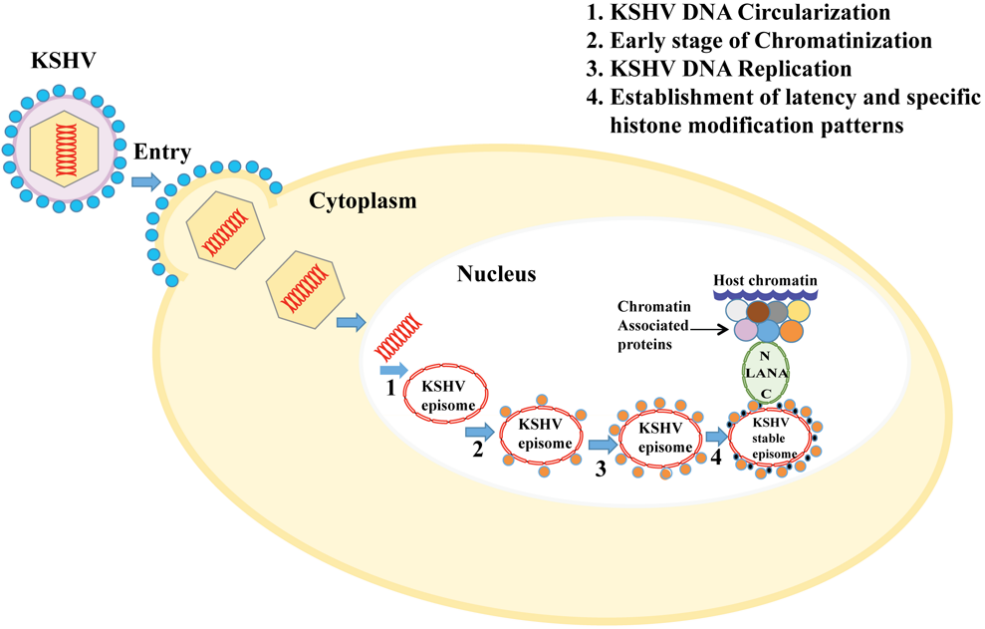
The chromatinization and maintenance of the KSHV genome following *de novo* infection: After the KSHV virion attaches to the host cell, the viral capsid enters the cytoplasm, followed by the ejection of viral DNA into the nucleus. Subsequently, the linear viral genome is circularized into an extrachromosomal episome to avoid detection by the host DNA damage response; (2) Circularized genome is further chromatinized using cellular histones and histone modifying factors resulting in a stable episome; (3) Viral genome, which is being maintained as multicopy chromatinized episome then replicates along with the cellular genome; (4) For the stable persistence and segregation of KSHV epigenome during latency, KSHV latent protein LANA binds to the TR region of the viral genome and tethers the viral genome to the host chromosome through its amino-terminus via interaction with histones and cellular chromatin associated proteins.

Each KSHV virion consists of a linear dsDNA duplex. The virion particle binds to the host cell surface receptor and penetrates into the host cell cytoplasm by a complex multistep process (reviewed in [52]). Virion capsids are then transported to the nuclear pore with concominant release of linear viral DNA. During *de novo* infection (*i.e*., entry of the virion in the target cells) and establishment of latency, the incoming linear dsDNA is circularized using the cellular enzymatic machinery to generate a closed-circular DNA, which is further maintained in a circular episomal form tightly packed as nucleosome (Figure 1). Encapsidated virion DNA is generally naïve and shows no chromatin structure, however, the resulting circular episome gets chromatinized due to its association with cellular histones in order to ensure: (1) protection of viral DNA ends to escape the host DNA damage response; (2) stable maintenance, replication and segregation of the viral genome to daughter cells during mitosis; (3) successful completion of viral life cycle; and (4) the regulation of viral gene expression (reviewed in [53]).

Though the processes of viral genome circularization and chromatinization are poorly understood, there are key regulatory steps in the life cycle of many viruses, which are crucial for the establishment and persistence of the viral quiescence or latency [53,54]. The viral genome upon entry into the host cell nucleus must adopt a structure similar to the host genome and interact with cellular chromatin. The chromatinized viral DNA is influenced by the same epigenetic factors as the cellular DNA thereby generating the so-called “*viral epigenome*”, which significantly impacts both latency and lytic reactivation of the viral genome. Additionally, studies have indicated that viruses that enter and hide in the host nucleus have co-evolved with numerous cellular chromatin modulation mechanisms to ensure their survival and propagation [54].

Recent studies have uncovered a wealth of information regarding the dynamic aspects of chromatin structure and the mechanisms whereby chromatin modifications regulate the viral gene expression and viral-host chromatin interactions. Importantly, chromatin and epigenetic modulation of KSHV genome represents a novel antiviral target for blocking virus-mediated tumorigenesis. This compilation review summarizes some of the emerging concepts that will describe in the detail our current knowledge of chromatin assembly and remodeling factors, and epigenetic alterations, including DNA methylation, post-translational histone modifications, and nucleosome occupancy of KSHV genome, as the master controllers of KSHV’s biphasic life cycle, gene expression pattern and associated pathogenesis.

## 2. Chromatin Regulation and Gene Expression Pattern

The eukaryotic DNA is wrapped around histone proteins to form nucleosomes, the fundamental repeating units of chromatin. The nucleosome consists of an octameric histone core containing two H2A-H2B dimers and one H3-H4 tetramer with 147-bp segment of DNA wrapped in 1.65 turns and ~50 bp of free DNA separating the neighboring nucleosomes [55]. Together with linker histone H1, nucleosomes can compact to form condensed chromatin. The histone tails that protrudes out from the nucleosome undergoes different posttranslational modifications such as lysine acetylation, lysine and arginine methylation, serine, tyrosine and threonine phosphorylation and lysine ubiquitination to provide coding mechanism for protein recognition and signaling (reviewed in [56,57]).

Acetylation of histone tails, carried out by histone acetyltransferases (HAT’s) results in the relaxation of the chromatin structure through increased charge repulsions and is primarily associated with active gene expression [58]. Higher-order folding of the nucleosomal DNA can give rise to either less condensed, *actively transcribed euchromatin* or to highly condensed, *transcriptionally silent heterochromatin* and histone modifications can be found in both varieties of the chromatin. Euchromatin state is characterized by high levels of histone acetylation and methylation of lysine residues at 4, 36 and 79 of histone H3. Alternatively, the heterochromatin has low levels of acetylation and high levels of methylation of lysine residues at 9 and 27 of histone H3. Genes that are transcriptionally active possess high levels of H3K4me3, H3K27ac, H2BK5ac and H4K20me1 histone marks in their promoter region and H3K79me1 and H4K20me1 along the gene body [59]. These patterns of histone modifications that trigger activation or silencing of the gene can potentially be transmitted to the daughter cells and thus referred to as sequence-independent heritable changes of the genome (*epigenome*). Several well defined “*epigenomic marks*” on chromatin range from chemical modifications on DNA, histone proteins and 3D chromatin organization.

Structure of chromatin is highly variable and dynamic and their structures vary among different cell types. Analysis of the chromatin structure at the genomic level leads to characterization of the landscape of the epigenome that helps to define the gene expression pattern of any cell. Advances in next-generation sequencing technologies have enabled a high-resolution genome-wide investigation of the viral and cellular epigenomic landscape in various cell types. Powerful sequencing-based methods are used as excellent tools to interrogate the interplay between genomic locations of open chromatin regions, DNA-binding factors, nucleosomes and chromatin conformations at a single nucleotide resolution [60,61].

Chromatin Immunoprecipitation (ChIP) analysis of DNA-histone complexes is one of the efficient methods for the elucidation of chromatin structure and histone modifications. ChIP assays combined with DNA microarrays (ChIP-on-Chip) or high-throughput sequencing of purified DNA fragments (ChIP-seq) and chromatin conformation capture assays (3C) are primarily used to characterize endogenous chromatin structure together with mapping the genomic locations of DNA-associated proteins. In the context of the KSHV genome, prior studies conducted on KSHV infected cell lines at nucleotide resolution using ChIP-seq established the presence of histones, DNA methylation, DNA-protein interactions such as RNA polymerase II binding, LANA and CTCF-cohesin occupancy and nucleosome depletion on the latent KSHV episome [62,63,64]. More recently FAIRE (Formaldehyde-Assisted Isolation of Regulatory Elements) has been utilized as an alternative and robust approach than antibody-dependent ChIP-sequencing, to depict several regions of open chromatin in the latent KSHV genome. FAIRE, followed by next-generation sequencing (FAIRE-seq), together with previously identified nucleosome depletion and histone modification data, has led to the identification of the regulatory elements in KSHV genome, adding to our existing knowledge on the whole genome landscape of latent KSHV chromatin [65].

## 3. KSHV Latent Gene Expression Profile

During latent infection, the KSHV genome persists in a relatively quiescent state as multicopy chromatinized “*minichromosomes* or *episomes*” that transcribes and replicates simultaneously with the cellular chromosome thereby evades host’s immune surveillance [66]. Major latent genes are located between ORF 69 and K14 in the KSHV genome in the latency locus with their gene expression under the control of a few promoters, whereas the other viral genes are silenced during latency (reviewed in [67]). Viral latency is, however, reversible and can be periodically reactivated into lytic replication by specific environmental and physiological stimuli, thereby expressing all of the viral lytic genes in an ordered cascade resulting in the production of viral progeny. Both latent and lytic cycle replications are essential for the long-term persistence of the virus, and the gene products of these transcriptional programs contribute to KSHV-induced pathogenesis [49,67].

Majority of the KS biopsy samples and PEL cell lines exhibits latent gene expression profiles, thus provides a remarkable tumor model for better understanding of the mechanisms that regulate the expression of viral genes during latency. These latency models express a restricted yet variable pattern of viral latency-associated genes that modulate both viral and cellular gene expressions to establish and maintain latency. KSHV latent genes or transcripts include the latency-associated nuclear antigen LANA encoded by ORF73, viral cyclinD homologue, v-Cyclin encoded by ORF72, a viral homologue of a FLICE-inhibitory protein v-FLIP encoded by ORF71, Kaposin encoded by K12, and a cluster of 12 viral microRNAs [68,69,70,71,72], mRNA of several other viral genes including viral G protein-coupled receptor v-GPCR encoded by ORF74, K1, vIL-6 and ORF59 encoded by ORF59 have also been detected in most KS and PEL-tumor models [14]. Together, these latency-associated viral proteins are required for constant latent infection and survival of the infected cell.

For most of the KSHV positive PEL-derived cell lines studied so far the expression of the major viral latent proteins: LANA, v-Cyclin and v-FLIP is limited to a single multi-cistronic latent transcript [66]. LANA, functional orthologue of Epstein-Barr virus encoded EBNA1, binds to multiple sites on the KSHV genome, preferentially in the TR region, which helps in tethering of the viral genome to the host chromosome and maintenance of the replicated episomal copies after duplication of the tumor cells during latency [73]. The v-Cyclin and v-FLIP play key roles in host cell proliferation and survival [74,75,76]. The Kaposin transcript is expressed either from promoters downstream of LANA or the ones within the repeat region upstream of Kaposin [77]. Along with ORF74, K14 is expressed as a bi-cistronic transcript from the promoter downstream of LANA-v-Cyclin-v-FLIP RNA [78]. The ORF74-K14 transcript initiates from the 5' UTR of the LANA-v-Cyclin-v-FLIP and is expressed in latently infected cells along with LANA-v-Cyclin-v-FLIP gene expression [79]. More sensitive methods using micro-arrays and proteomics have identified several viral transcripts and peptide motifs that further provide valuable knowledge regarding viral latent gene expression [80].

*Latency-associated Nuclear Antigen (LANA):* LANA, a large nuclear protein of 220–230 kDa with DNA binding and chromatin binding domains in carboxy and amnio terminal domains, respectively is encoded by ORF73 and is critical for the establishment of latent infection [73,81,82]. LANA, a multifunctional protein can regulate transcription of cellular and viral genes through activation or repression of various cellular and viral promoters [83]. Additionally, LANA has also been shown to deregulate the expressions many oncogenes (by stabilizing) and tumor suppressors (by degrading) to induce tumor growth and these includes c-Myc, p53, hypoxia-inducible factor 1 (HIF-1), glycogen synthase kinase 3 (GSK3), von Hippel-Lindau protein (pVHL) and β-catenin [84,85,86,87,88,89]. LANA also controls lytic origin dependent DNA synthesis by interacting with origin binding protein (OBP), K-bZIP [90]. LANA promotes tumorigenesis and cell growth by inducing chromosome instability and upregulation of cellular IAP expression in KSHV-infected cells [91]. LANA plays a critical role in maintaining the KSHV genome in infected cells by ensuring faithful segregation of the newly synthesized viral genome into the divided cells by interacting with cellular proteins, Bub-1 and CENP-F [92]. Additionally, LANA inhibits interleukin-4 (IL-4)-mediated STAT6 phosphorylation, which helps in blocking apoptosis to maintain latency [93].

The role of LANA in the persistence of the KSHV genome was identified by using the 33-kb left-end of KSHV in a persistence assay in BJAB cells expressing LANA [73]. Additional studies identified the LANA binding sites, named LBS1 in the TR region of the KSHV genome [81,94,95]. Minimal LANA binding sequence in TR was identified by *in vitro* binding assays to be a 13 bp sequence within the TR [95]. Additional studies identified another LANA binding site (LBS2), which has lower affinity than LBS1 and is located right next to the first LANA binding site (LBS1) in the TRs [96]. The first LANA binding site lies between positions 571 and 589 of the 801bp long TR and is termed as LANA binding sequence 1 (LBS1) [96]. Binding of LANA to these two LANA Binding Sequences (LBS1/2) of the TR creates DNA bending and suppresses transcriptional activities when fused to a reporter plasmid [94,97].

The DNA binding domain of LANA is mapped to the C-terminal domain between amino acids 996 and 1139 [98]. Scanning of this region for identifying the exact residues in DNA binding by generating substitution mutants determined that amino acids between 1007 and 10021aa may be the DNA contact residues as the mutants of this region abolished DNA binding as well as replication and episome persistence abilities of LANA [98]. The amino terminal domain of LANA binds to the host chromatin through nucleosomal histones and tethers the viral genome bound to the C-terminus of LANA to the host chromosomes. Along with histones various other chromatin-associated proteins including RING3, which colocalizes to heterochromatin and heterochromatin proteins 1 (HP1) was also detected as LANA binding proteins by various biochemical assays [99]. A model for tethering of LANA to the host chromatin either through N-terminus binding proteins such as linker histone H1, core histones H2A/H2B, budding uninhibited by benzimidazole 1 (Bub1) and centromeric protein F (CENP-F) or C-terminus binding proteins including 43-kDa protein DEK, methyl CpG binding protein (MeCP2), bromodomain proteins Brd2/Brd4, nuclear mitotic apparatus protein CENP-F and Bub1 has been proposed [100].

A number of studies have demonstrated the role of LANA in modulating the activities of various cellular pathways by directly interacting with the major players of the pathways or complexing with additional proteins to favor cell growth (reviewed in [101]). LANA does not seem to possess enzymatic activities required for DNA replication but is critical for the replication of TR-containing plasmids [102]. LANA achieves this by recruiting host cellular replication factors to the TR in a cell cycle dependent manner (reviewed in [101,103]. Studies from others as well as our lab have shown the involvement of host cellular replication proteins in the replication of KSHV episomal DNA [104,105,106,107]. The replication of host genome occurs in a very precise manner in order to maintain genetic integrity, which is achieved by ensuring that no segment of DNA replicates more than once per cell cycle [108]. Replication process starts during the late G_1_ and early S phase by licensing of the replication origin sites by sequential loading of ORCs, cdc6, Cdt1, and the heterohexameric complex MCM2-7 (minichromosome maintenance proteins 2 to 7) to the origins to be used during the S phase [109]. Upon loading of these licensing factors, origins are licensed to form a pre-replicative complex (pre-RC) for replication initiation [107,110].

LANA has also been shown to upregulate the proteins important for the immortalization of infected cells, which includes modulation of hTERT and E2F1 responsive promoters [111,112]. LANA also promotes cell cycle progression through redistribution of the cellular pool of glycogen synthase kinase-3 beta (GSK-3b) to the nucleus, which leads to an accumulation of β-catenin in the cytoplasm. Increased levels of β-catenin in the cytoplasm are translocated to the nucleus, which in turns upregulates the β-catenin responsive, Lef-Tcf promoters. Increased levels of Lef-Tcf enhance the transcription of S-phase entry genes such as *MYC*, *JUN and CCND1* (Cyclin D1), which drive cell cycle progression [85]. Additionally, LANA modulates apoptotic pathways as the LANA expressing cells are resistant to p53-dependent apoptosis but not the p53-independent apoptosis confirming specificity to p53 [112]. LANA also modulates the activity of another tumor suppressor, pRb, which was determined by the fact that expression of LANA overcame the flat-cell (growth arrested) phenotype in RB1 negative cells. Saos2 (deletion mutant of *RB1*) cells enter cell cycle arrest upon exogenous expression of pRb, but the interaction of LANA with pRb overcomes this phenotype, suggesting re-entry of these cells into the S-phase of cell cycle. LANA expressing cells inhibits pRb and p53 pathways that enable these cells to circumvent the G1/S checkpoint and the apoptotic pathway, respectively, which leads to the immortalization and tumorigenesis of LANA expressing cells [113].

v*-Cyclin:* It is encoded by ORF72 and is the homologue of cellular D-cyclin which acts like a constitutive activator of cellular cyclin-dependent kinase 6 (CDK6) to regulate cellular proliferation and viral replication [114]. The v-Cyclin-CDK6 complex can phosphorylate its cellular counterpart pRb protein, Histones H1, CDK inhibitor (cdki) and p27 (Kip1) [115]. The exact role of this viral protein in regulating KSHV life cycle is not fully understood but studies indicate that v-Cyclin-CDK6 complex mediated phosphorylation of nucleophosmin (NPM) facilitates NPM-LANA interaction and recruitment of HDAC1 to promote KSHV latency [116]. Additionally, KSHV v-Cyclin shares close functional relationship with murine gammaherpesvirus 68 (MHV68) v-Cyclin that is known to mediate efficient lytic reactivation from latency [117].

v*-FLIP/K13:* ORF71 encodes the KSHV homologue of cellular FLICE like inhibitory protein v-FLIP, also known as K13. KSHV v-FLIP is likely to contribute to latency by promoting cell survival [74,118]. The v-FLIP can block apoptosis by binding to the inhibitor of kB-kinase γ (IKK γ), leading to the activation of the NF-*k*B pathway [119]. NF-*k*B pathway activation by v-FLIP has been linked to KSHV lytic replication as the KSHV mutant deficient in v-FLIP inhibits ORF50/RTA lytic gene expression [120]. Additionally, v-FLIP regulates the activation of a key cellular survival pathway to promote cell proliferation and survival during latency (reviewed in [121]).

*Kaposins*: The Kaposin locus (K12) and surrounding direct repeat regions DR1 and DR2 encode for 3 proteins, namely, Kaposin A, B and C [122]. Kaposin A is a hydrophobic latent protein with transforming potential in Rat-1 fibroblasts whereas Kaposin B is a small soluble nuclear protein, which affects signaling by binding to a mitogen-activated protein (MAP) kinase 2 (MK2) [123]. Kaposin C is a trans-membrane protein, however the biological function of this protein is not yet known [122]. All of these proteins are shown to contribute to the inflammatory microenvironment of KS [122].

*LANA2/*v*IRF-3*: The KSHV genome encodes four viral homologues of cellular interferon regulatory protein (vIRFs) to counteract the interferon system (IFN) for evading the host’s immune response. One such protein predominantly expressed during KSHV latency is K10.5/LANA2 that is considered as a KSHV defense protein against IFN [124]. LANA2 is exclusively expressed in both PEL and MCD cell lines and has been linked to the disruption of cellular IRF-7, IRF-3 and IRF-5 mediated signaling and cellular PKR signaling [125]. LANA2 also inhibits p53 and NF-*k*B, which suggests its role in KSHV infection and pathogenesis [126].

*Viral microRNAs*: KSHV encodes 12 pre-miRNAs, of which 10 miRNA (miR-K1-9 and -K11) are encoded between the kaposin and ORF71/K13 region while the other two miRNAs, miR-K10 and -K12 are located in the coding and 3' untranslated region of K12, respectively [127,128,129,130,131]. All miRNAs are expressed in cells latently infected with KSHV and regulate latency while suppressing lytic reactivation of the virus [132,133,134,135]. Among these miRNAs, miR-K1 represses the expression of IκBα-an inhibitor of the pro-survival NF-κB pathway and inhibits the activation of lytic viral promoters. Since the NF-κB pathway is involved in cell growth and survival, miR-K1 might directly affect KSHV-mediated pathogenesis [136,137]. KSHV miR-K10 targets TNF-like weak inducer of apoptosis (TWEAKR) and is shown to protect cell from apoptosis and suppress pro-inflammatory responses, which might contribute to KSHV latent infection [136,138]. Several other miRNAs such as miR-K3, -K4, -K5 and -K9 target nuclear factor I/B, Rbl2 protein, Bcl-2 associated factor, BCLAF1 and activates the 3'UTR region of RTA protein respectively, to inhibit cell death and regulate lytic replication [139].

### Chromatin Organization of KSHV Genome during Latent Infection

*LANA-dependent DNA Replication and Epigenetic Modifications at the KSHV TR*: KSHV LANA, a DNA binding nuclear protein acts as an initiator of DNA replication by specifically binding to a ~60 bp motif in the KSHV TRs [102]. A DNA-LANA protein complex is formed between LANA, trans-acting protein, and the LANA binding, cis-elements of KSHV TR [94,95,140]. Two or more copies of the TR are required for maintenance of the plasmid while a single copy of TR and the minimal replicator element is sufficient to initiate DNA replication [98,107,141,142]. Despite the absence of any enzymatic activity in LANA required for DNA replication, LANA supports DNA replication via its interaction with the host cellular replication factors including the origin recognition complexes (ORCs), Topoismerase IIβ, replication protein A (RPA) and proliferating cell nuclear antigen (PCNA) [104,106,110]. A recent study has demonstrated that LANA mediates KSHV DNA replication and virus persistence by recruiting replication factor C (RFC), the DNA polymerase clamp, proliferating cell nuclear antigen (PCNA) loader. LANA mutant with deleted RFC binding domain was found to have a negative impact on LANA-mediated DNA replication and episome persistence [110]. However, the detailed mechanism by which LANA interacts with ORCs and other replication proteins to initiate replication needs to be explored. Although, the LANA-dependent TR replication is believed to be the primary site of replication initiation but, KSHV DNA synthesis can also occur outside of the TR by the autonomously replicating element in the long unique region (LUR) of the viral genome.

During the G_1_/S transition phases of the cell cycle, chromatin structure at the TR (nucleosomes and two LANA binding sites) is modified to make the viral DNA more accessible to the host cell DNA replication machinery. Two independent new studies [143,144] have utilized chromatin immunoprecipitation data together with KSHV specific microarrays (CHIP-on-chip) to generate high-resolution profiles of the chromatin structure of the entire KSHV episomes in latently-infected cell lines, providing novel genome-wide sequencing of the epigenetic landscape of KSHV genome. Characteristic DNA methylation patterns were found throughout the KSHV genome, though KSHV is not subjected to extensive DNA methylation. Various peaks of CpG methylation were observed with remarkable similarity among different cell lines, ranging from PELs and infected SLKs. In all cases, regions immediately upstream of LANA and several locations including K9, ORF45/50, K7 and ORF8 were deprived of CpG methylation [143,144].

Studies so far clearly show that latent KSHV genome is extensively chromatinized with both the activating (H3ac and H3K4me3) and repressive (H3K9me3 and H3K27me3) histone marks among them the Latency-associated genes possess activating histone marks as well as colocalize with transcriptionally active RNA polymerase II (RNAPII). Immediate early (IE) and early genes (E) have bivalent chromatin marks (acH3, H3K4me3 and H3K27me3) or active chromatin marks (acH3/H3K4me3-rich) whereas all of the late genes have H3K9me3- and H3K27me3-marked heterochromatin. Also, two of the chromatin modifying enzymes: EZH2 (H3K27me3 histone methyltransferase) of the Polycomb Repressive Complex 2 (PRC2) family and JMJD2A (H3K9me3 histone demethylase) have been shown to associate with the latent KSHV genome [143,145,146]. EZH2, which ubiquitously binds on the viral genome represses the lytic gene expression program during latency and JMJD2 that binds to the acH3/H3K4me3 chromatin regions of KSHV genome, guards the methylation of H3K9. Overall, the whole KSHV genome epigenetic analysis indicates that KSHV genome chromatinization regulates the expression of latent and lytic genes in a systematic manner.

**Figure 2:**
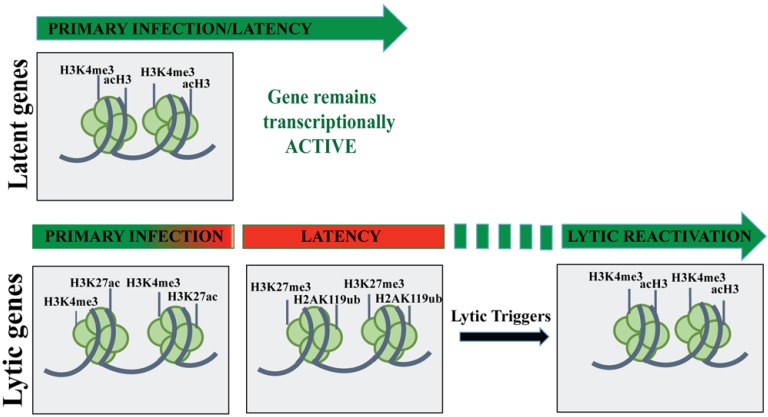
The chromatin landscape of the KSHV genome during various phases of viral life cycle. Following primary infection, promoters of the key latent genes get epigenetically modified with activating histone marks (H3K4me3/acH3) resulting in the expression of those genes throughout the viral life cycle. In contrast, KSHV lytic gene promoters possess either H3K27Ac/H3K4Me3-rich, active chromatin or H3Ac/H3K4Me3/H3K27Me3-rich bivalent chromatin. During latency, the lytic gene promoters are epigenetically modified with H3K9Me3/H3K27Me3 marks to form a heterochromatin. However, upon reactivation these repressive histone marks are enzymatically removed and gets modified with the activating histone marks (H3K4Me3/H3Ac).

Additionally, the DNA replication is governed by various epigenetic modifications including DNA methylation, post-synthesis histone modification patterns and nucleosome occupancy [147]. DNA methylation slowly builds up over the entire KSHV genome following the *de novo* infection and typically represses the viral gene expression [148] (Figure 2). For KSHV, DNA methylation does not exist at transcriptionally active LANA promoters, however it is found at several transcriptionally silent regions [53]. Though the factors governing the locus-specific methylation of DNA are not clearly understood, it is predicted that some sites are methylated due to a lack of transcriptional activity whereas others lack DNA methylation due to the inhibitory effects by DNA-binding proteins. Chromatin immunoprecipitation assays suggest that KSHV TR is associated with stable positioned nucleosomes that are further subjected to cell cycle regulation and nucleosome remodeling [104].

*CTCF-cohesin Binding and Higher-order Chromatin Conformation*: The association of architectural proteins, cellular chromatin boundary factor (CTCF) and cohesin plays an integral role in sister-chromatid cohesion and chromosome segregation during mitosis [149]. CTCF/cohesin binds to the first intron of the LANA-vCyclin-vFLIP multicistronic latent transcript in KSHV, nearly 15 kb from the TR [150]. Depletion of the CTCF, cohesin or the CTCF-cohesin binding sites in the KSHV bacmid leads to dysregulated gene expression [150,151]. This suggests that CTCF-cohesin complex provides a chromosome-organizing center that facilitates stable episome maintenance [150]. Genome-wide ChIP analysis combined with next generation sequencing of the KSHV genome indicate cohesin binding sites are colocalized with CTCF binding sites at several sites throughout the latent KSHV genomes [62,150]. These studies support that the DNA-binding factor CTCF/CTCF cluster is important for the control of latent transcription. A recent study further demonstrated that CTCF and cohesion binding on the viral genome dynamically changes during viral reactivation and depletion of these chromatin insulators positively regulate the transcription of lytic genes [152]. The molecular functions of these CTCF-cohesin clusters are beginning to understand and it is likely that during mitotic cell division these clusters correlate with chromosome for segregation and gene transcription [153].

Higher order DNA structure is likely to be a heritable epigenetic regulatory factor. Higher order DNA structures like those involving DNA looping have been identified for the CTCF binding sites in the KSHV LANA promoter region [154]. A loop was formed with the 3' end of the KSHV latency transcripts ending at the K12 gene suggesting that the entire latency transcription area is restrained to a DNA loop mediated in part by CTCF binding. Using the viral genome-wide chromatin conformation capture method (3C), chromatin conformation of the latent EBV and KSHV genomes has been depicted recently and CTCF/cohesin binding sites have been found to be physically linked to other regions of the viral genomes *via* extensive DNA-loop formation [150]. For KSHV, a long DNA loop is found between the LANA and RTA control regions and these loops are stabilized by cohesin subunits primarily SMC1, SMC3 and Rad21 and SA1/SA2 [62]. Depletion of cohesin subunits disrupts the DNA loop between the latency and lytic promoter regions and a robust reactivation of lytic cycle gene transcription, indicating an important role of chromatin organizing factors and chromosome conformation in maintenance of stable gene programs during KSHV latent infection [150].

Recently, MAPit (Methylation Accessibility Probing for individual templates) single-molecule footprinting assays were employed to characterize several chromatin states at selected loci within mammalian nuclei [155,156]. The chromatin structures of the latency promoter and immediate early lytic genes, RTA and K2 promoter were investigated. The results indicated a heterogeneous chromatin structure with both fully closed and open conformations being present at investigated promoter regions in the KSHV genome. In addition, the epigenetic drift, *i.e.*, imperfect maintenance of the chromatin states was found to coordinate the latent and lytic gene control [156].

## 4. KSHV Lytic Gene Expression Profile

Within the predominantly latent population of KS spindle cells a few percent of the latent cells express markers of lytic replication [157,158]. During lytic DNA replication, a linear form of dsDNA molecule is generated and one copy of viral DNA is packaged per virion. For KSHV, viral lytic genes promote cell proliferation, survival and angiogenesis leading to onset and progression of KS lesions [15,159].

During lytic phase, viral gene expression is time-controlled and tightly regulated in order to allow a systematic synthesis of viral gene products. Lytic genes are widely distributed across the whole KSHV genome with their expression been controlled by several different promoters. Genes expressed during the lytic cycle can be grouped according to their timing and expression in response to the protein synthesis/DNA replication inhibitors as immediate early (IE), early (E) and late (L) genes [160,161,162,163]. IE genes include RTA/ORF50, ORF45 and K4.2, the primary genes expressed during lytic replication and encode regulatory proteins for viral replication. The latent to lytic switch of KSHV is regulated by the Replication and Transcription Activator, RTA, a 110 kDa protein, encoded by the ORF50 gene, which is capable of inducing the cascade of lytic gene expression including viral microphage inflammatory protein-I, viral interleukin 6 (vIL-6), ORF59, ORF65 and K8.1 and the production of DNase-resistant encapsidated viral DNA. Interestingly, the over-expression of RTA protein alone is necessary and sufficient to disrupt KSHV latency and initiate the lytic replication cascade [164,165,166,167]. Many early genes (E) have enzymatic functions (ORF59), regulation of gene expression (MTA protein), modulation of the immune system (MIR1/2) and selective accelerated turnover of host mRNA (ORF37) [168,169,170]. The expression of early genes (E) is controlled by the IE genes and include the polyadenylated nuclear RNA (PAN RNA), Kaposin, ORF57, k-bZIP (K8), K5, K9, K14, K15 ORF6, ORF21 and ORF74 [15], followed by expression of the late genes including major capsid protein (MCP) encoded by ORF25 and the small viral capsid (sVCA). Late genes are transcribed following DNA replication and they are the structural genes for virus assembly [160,161].

Recently, anem’s group performed a comprehensive genome analysis of transcriptional and translational profiles of the KSHV genome during the productive/lytic infection in epithelial iSLK-219 cell line using a combination of mRNA-sequencing (mRNA-seq) and ribosome profiling (Ribo-seq) [171]. This showed that ribosomes promptly bind to the transcripts of lytic genes during reactivation, suggesting that they are regulated at the transcriptional level. These modern approaches also revealed a wealth of additional information including occupancy of ribosomes on viral non coding RNA, numerous small open reading frames (ORFs), alternative splicing sites and alternative translation initiation sites to expand the coding potential of the viral genome. These new features of the KSHV genome led to a new annotation, which is termed, KSHV 2.0 [171].

### 4.1. Chromatin Organization of the KSHV Genome during Lytic Reactivation

The Lytic reactivation is regulated by alteration in the histone modifications of the viral genome including acetylation of core histone tails by histone acetyltransferases (HATs) and making the chromatin transcriptionally active. On the other hand, deacetylation of histone tails by histone acetyltransferases (HDACs) condenses the chromatin to make it transcriptionally silent [172]. During latency, HDACs attach to the RTA promoter resulting in hypoacetylation of histones and an inactive promoter [173,174]. However, the RTA promoter can be activated by physiological conditions, such as hypoxia, or in latently infected cell cultures by treatment with HDAC inhibitors, sodium butyrate (NaB) or the HAT inducer tetradecanoylphorbol acetate (TPA) leading to hyperacetylation of histones [175]. Additionally, DNA methylation plays an important role in controlling lytic reactivation as the inhibitor of enzyme DNA methyltransferases, 5-azacytidine (5-AzaC) triggers the KSHV lytic cycle [176]. The KSHV genome is subjected to methylation at CpG dinucleotides and shows CpG suppression at the RTA promoter during latency [144]. Together, these facts indicate that epigenetic modifications play an important role in the lytic reactivation process.

*KSHV viral long non-coding PAN RNA and gene regulation*: Among the early gene transcripts, PAN RNA is the most abundant lytic cycle transcript of KSHV [158,161]. New insights suggest an important role for long non-coding RNAs (lncRNAs) in the regulation of gene expression patterns via modulation of the lytic switch [177]. Long non-coding RNAs often referred to as “junk-regions” are RNAs which are typically longer than 200 bases that do not code for any protein [178]. KSHV expresses an unusual ncRNA called Polyadenylated nuclear non-coding RNA, abbreviated as PAN RNA in the nucleus during lytic induction that down regulates the expression of many immunomodulatory genes [176,179]. PAN RNA is transcribed by Pol II, is capped at its 5' end and ends with a 3' polyadenylate tail [180]. The expression of PAN RNA appears to be tightly regulated by RTA through a *cis*-acting RTA Response Element (RRE) present in the PAN RNA promoter region and by ORF57, which further stabilizes PAN RNA [181,182,183]. Although PAN RNA interacts with several viral and cellular encoded proteins to suppress gene expression, the exact role of PAN RNA in virus replication and KSHV growth is yet to be elucidated owing to the difficulty of generating a recombinant virus lacking the PAN RNA.

**Figure 3:**
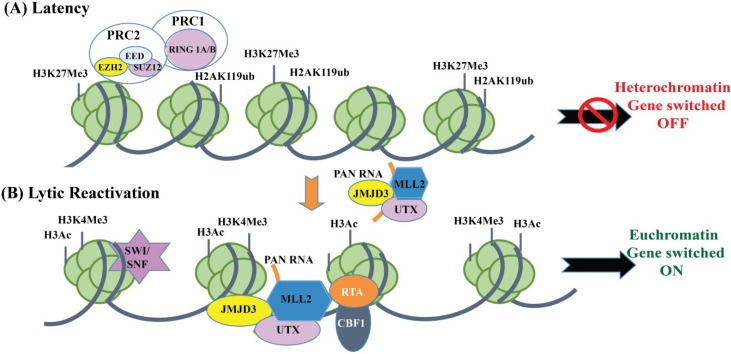
Polycomb repressive complexes (PRC1 and PRC2) and Polyadenylated RNA-mediated control of KSHV RTA promoter during latency and lytic reactivation. (**A**) During latency, the histone methyltransferase EZH2 subunit of PRC2 and RING 1A/B subunit of PRC1 bind to the KSHV genome and colocalizes with corresponding repressive histone marks H3K27me3 and H2AK119ub, respectively, leading to silencing of the target genes; (**B**) Lytic reactivation, triggered by the dissociation of PRC2 and PRC1 from the viral genome or inhibition of EZH2 by chemical compounds, leads to the enrichment of activating histone marks on the viral genome and the activation of gene transcription. PAN RNA, which is highly expressed during lytic reactivation, recruits several cellular and viral chromatin factors (MLL2, JMJD3 and UTX) to the RTA promoter and triggers the expression of immediate early, RTA protein.

In order to elucidate the role of PAN RNA in the KSHV life cycle, recent studies on a recombinant BACmid with a deleted PAN RNA locus showed decreased RTA expression in the induced cells at both the early and late induction time points [177]. The RTA promoter is enriched in both activating (H3K4me3) and repressive (H3K27me3) histone marks. As a result, the chromatin of RTA promoter is often referred to as a bivalent promoter [144]. H3K27me3 is deposited by one of the Polycomb-group proteins, namely Polycomb Repressive Complex 2 (PRC2) that consists of subunit: EZH2, SUZ12, EED and the histone binding proteins RbAp48/46. Upon reactivation, chromatin remodeling proteins, JMJD3 and UTX H3K27me3 histone demethylases and H3K4me3 histone methyltransferase are recruited to the RTA promoter via KSHV-encoded Polyadenylated nuclear non-coding PAN RNA that disrupts polycomb mediated chromatin repression [177]. RTA binds to its own promoter through the cellular transcription factor CBF1 and recruits histone acetyltransferases (CBP/p300) and chromatin remodeling factors (SWI/SNF2) to modify the viral chromatin structure to a transcriptionally active state allowing a complete cycle of viral reactivation (Figure 3). RNA ChIP assays show that PAN RNA interacts with histone H3K27 demethylases JMJD3, UTX and methylases MLL2 to reverse the Polycomb-mediated repression of viral IE RTA transcripts through an interaction with the viral genome [177]. These studies further establish that PAN RNA is a multifunctional regulatory transcript that controls KSHV gene expression by mediating chromatin-modulations of the KSHV repressed genome.

### 4.2. Chromatin Organization of KSHV Genome during de Novo Infection

PEL cell lines are latently infected by KSHV and can be induced to trigger lytic cycle and produce infectious virions. However, PEL cells being lymphoid in nature do not support *de novo* KSHV infection, viral replication or serial propagation. Similarly, many other standard established cell lines such as 293 cells have been reported to support a very low level of KSHV infection with limited release of infectious virus [184,185]. These limitations have impacted the development of systems for the genetic analysis of KSHV and in turn a deeper understanding of early events of KSHV infection.

The process of KSHV *de novo* or primary infection involves attachment of the viral envelope proteins to host cell receptors and entry in the target cells by a multistep complex process [186]. Several transmembrane glycoproteins, encoded by KSHV have been found to be involved in the attachment and entry of KSHV in the target cells including gB (ORF8), gH (ORF22), gL (ORF 47), gM (ORF39) and gN (ORF53) [187,188,189]. Following the entry of viral genome into the nucleus, the viral genome undergoes extensive modification including circularization and chromatinization to escape the host cell defenses [53,190]. KSHV encoded ORF75 protein, which belongs a formylglycineamide ribotide amidotransferase (FGARAT), blocks cellular defenses by antagonizing the ND10 (nuclear domain 10) components [190]. Since chromatin association of the genome restricts the access of transcription factors to the viral promoter regions, modifications of the viral chromatin play an integral role in controlling viral gene expression [191].

Two independent studies have used ChIP-on-chip assays to provide the first unbiased and genome-wide views of the latent KSHV chromatin in infected BCBL-1 and SLK cells indicating a uniform distribution of total histone H3 and genomic localization of its modified forms, *i.e.*, activated H3K4me3 and acetylated H3K9 (acH3) and repressive histone marks [143,144]. Latent genes, IE and E lytic genes are found to be rich in H3K4me3/H3K9 (acH3) during latency and reactivation whereas the genomic regions of KSHV that encode for many late lytic genes display high levels of H3K9me3 /H3K27me3 during latency and early lytic reactivation [67].

In order to provide an overall description of the pre-latency phase of KSHV infection, a comprehensive epigenetic study was performed by Dr. Jae Jung’s group on SLK cells using ChIP-on-chip and FAIRE analysis [192]. During the onset of infection, the KSHV epigenome develops a transcriptionally active chromatin structure (euchromatin) with a high level of activating histone marks H3K4me3 and H3K27ac, accompanied by the temporary induction of a limited number of lytic genes, which further increase the activating histone marks on the viral genome. Between 24 and 72 h post-infection, levels of activating histone marks decrease due to the Polycomb group protein (PcG protein)-mediated increase in the amount of repressive histone marks H3K27me3 and H2AK119ub, thereby inhibiting lytic gene expression. PcG proteins are cellular transcription silencing proteins that form enzymatic complexes with cellular Polycomb Repressive Complex 1 (PRC1) and PRC2 [193]. *De novo* acquisition of H3K27me3 marks is catalyzed by EZH2, which together with SUZ12 and EED form the subunits of the PRC2 complex. Both PRC1 and PRC2 are recruited to the KSHV genome leading to the temporally ordered biphasic euchromatin to heterochromatin transition in SLK and TIME cells, following *de novo* infection [192]. In contrast, KSHV is proposed to exist in a transcriptionally active euchromatin form in oral epithelial cells, resulting in efficient and robust lytic gene expression. Thus, the differential epigenetic modification of the KSHV genome in distinct cell types is a potential determining factor for latent infection *versus* lytic replication [192].

During the past years, components of distinct nuclear compartments called Promyelocytic leukemia nuclear bodies (PML-NB) have been found to regulate viral chromatin and viral gene expression [194]. PML-NB, also called nuclear domain 10 (ND10s) are nuclear multi-protein complexes that are 0.2–1.0 µm in size and include several subunits like Daxx (Death domain associated protein, Sp100 (speckled protein of 100 kDa), SUMO (small ubiquitin-related modifier) and 53 kDa protein associated with the nuclear matrix [195,196]. ND10s are the mediators of the innate antiviral defense mechanism and many viruses have developed strategies to counteract repressive properties of ND10s [196,197]. A systematic study by Grundhoff’s group reported the role of ND10s and its core components on the establishment of KSHV latency during the early infection phase in *de novo* infected SLK, established from tumor biopsy of oral mucosa, cells [148]. The KSHV episome/LANA in iSLK cells is found not to directly or transiently interact with ND10s during the establishment of latency (between 24 and 48 h post infection) *i.e.*, the time period when H3K27me3 marks accumulate on the KSHV episome. However, KSHV infection is reported to influence relocalization of ND10s components especially Sp100 protein, which is efficiently and permanently relocalized from nucleoplasmic and chromatin-associated fractions into the insoluble matrix by LANA, which induces SUMOylation of Sp100. Depletion of ND10s core components, Sp100, PML or Daxx did not interfere with latency establishment, though depletion of Sp100 accelerates the occupancy of the repressive histone mark H3K27me3 on viral episomes indicating that Sp100 acts as a negative regulator of PRC2 recruitment onto the KSHV genome [148].

## 5. Conclusions

KSHV is a complex and sophisticated oncogenic virus that developed numerous regulatory mechanisms to modulate the host-cell proliferation, apoptosis and host immune evasion, enabling the prolonged survival of the infected cell with the following latent infection and lytic reactivation. Despite the enormous wealth of information available about the mechanisms of how gammaherpesviruses persist in tumor cells, we are far from fully understanding the mechanism of latency establishment by KSHV, and the key cellular and viral factors that are responsible for restricted lytic gene expression during primary infection. Undoubtedly, the chromatin remodeling and epigenetic modifications of both viral and host genomes play crucial roles in determining the expression pattern of genes, that will lead to abortive or persistent infection. These epigenetic modifications include DNA methylation, histones post-translational modifications and higher-order chromosome conformations, including long intervening DNA loops. More importantly, prior to the chromatinization of incoming viral genomes, function of the cellular chromatin modifying enzymes is deregulated by a combination of viral proteins in order to re-program the cellular gene expression profiles and generate an altered chromatin landscape of KSHV genome favoring latency establishment [198,199]. Although the advances in technological development has enabled extremely high-resolution whole-epigenome analysis of KSHV on a large scale and provided us with a better picture of the chromatin regulation of the KSHV latent and lytic genomes, much remains to be discovered for understanding the complex orchestration of various epigenetic players that regulate KSHV chromatin remodeling by site-specific recruitment of histone-modifying machinery during various stages of viral infection. Therefore, further investigations of significant cellular and viral factors along with most important epigenetic regulatory factors, including histone-modifying enzymes, HDAC inhibitors and demethylating agents that alter the chromatin status of KSHV towards the formation of unique latent epigenomes need to be performed in order (1) to generate a detailed and complete epigenomic map for KSHV; and (2) to provide novel therapeutic strategies that can be exploited for controlling KSHV infection and KSHV-driven carcinogenesis.
